# Assessing the role of parent-child conflict and closeness in children’s depression: insights from a meta-analysis

**DOI:** 10.1186/s13034-025-00955-9

**Published:** 2025-09-29

**Authors:** Juanjuan Sun, Yuling Yin, Jinghui Zhang, Yan Li

**Affiliations:** Shanghai Institute of Early Childhood Education, Shanghai Normal University, 100 Guilin Road, Xuhui District, Shanghai, China

**Keywords:** Parent–child relationship, Depression, Children, Meta-analysis

## Abstract

Depression, as a prevalent public health concern, has long been the focus of research attention. However, the magnitude and moderating mechanisms underlying the association between parent–child relationships and childhood depression remain inconclusive. This study employed a meta-analysis to quantitatively assess the associations between two dimensions of parent–child relationships (closeness and conflict) and depressive symptoms in children across diverse global cultures, while investigating potential moderators through subgroup analyses and meta-regression. After systematic Literature search and screening, 63 studies comprising 97 effect sizes were included, with a total sample of 70,687 participants (mean age 13.3 ± 1.12 years; 51% girls). Main effect analysis revealed significant positive correlations between childhood depression and parent–child conflict (*r* = 0.25) and significant negative correlations with parent–child closeness (*r* = − 0.24). Moderator analyses identified cultural context, study design, child age, and publication status as significant moderators of these associations. In contrast, child gender, parental gender, and measurement instruments for depression showed no significant moderating effects. This study confirms that parent–child closeness and conflict respectively serve as crucial protective and risk factors for childhood depression. These findings underscore the importance of fostering positive parent–child relationships in preventive interventions, potentially reducing the incidence of childhood depression through improved family dynamics.

Depression is one of the most prevalent mood disorders in adolescence, characterized by persistent psychopathological features across developmental stages, typically emerging during puberty and continuing to exert influence into adulthood [1, 2]. Global Burden of Disease analyses by the World Health Organization [3] reveal that depression has become the leading cause of disability among individuals aged 15–29 years, with disability-adjusted life years (DALYs) attributable to depression increasing by 23% compared to 2020 levels [4]. Notably, this psychological crisis demonstrates a significant trend toward younger age of onset and Geographical heterogeneity. Empirical studies report depression detection rates of 12.8–18.1% among US adolescents [5], while the prevalence of depressive symptoms in Chinese pediatric populations has exceeded 10% [6]. Prediction models based on multinational cohort data indicate that adolescent depression incidence rates will increase by 34% from pre-pandemic baseline levels by 2024 [7]. Without effective intervention, depressive symptoms not only induce immediate cognitive impairment and reduced social participation, but also exert enduring impacts on psychosocial functioning from adolescence through adulthood [8, 9].

Positive interpersonal relationships contribute to mitigating and inhibiting depression [10]. Bronfenbrenner’s ecological systems theory [11] posits that the family, as the central microsystem, establishes foundational influences on the formation of children’s emotional regulation mechanisms through parent–child relationship quality. Furthermore, parent–child interactions not only shape adolescents’ emotional schemas through daily parenting practices [12] but also modulate the neurodevelopmental adaptability of stress response systems [13]. Cross-cultural studies corroborate that children with secure parental attachments develop more sophisticated stress-coping strategies, demonstrating a 42% reduction in depression incidence attributable to neuroplasticity differences [14, 15]. While existing research has identified multiple pathways linking parent–child relationships to childhood depression, significant discrepancies persist in reported effect magnitudes. For instance, longitudinal studies document predictive effect sizes of parent–child relationship quality on depression ranging from − 0.09 [16] to − 0.54 [17]. The methodological strength of meta-analysis lies in its capacity to quantify aggregated effect sizes across heterogeneous studies while identifying critical boundary conditions (e.g., cultural contexts, developmental stages) through moderator analyses that explain effect heterogeneity. Building on this framework, the present study will employ meta-analysis to elucidate the association mechanisms between parent–child relationships and childhood depression, thereby informing evidence-based strategies for enhancing mental health and developmental well-being.

## Parent–child relationship and children’s depression

Childhood depression is a mood disorder characterized by persistent low mood, diminished interest, somatic symptoms (e.g., sleep disturbances, changes in appetite), and cognitive impairment (e.g., distraction, self-denial) [18] which progresses with developmental stage and encompasses depressive mood, depressive symptoms, and clinically diagnosed depressive neurosis (defined by chronic, less severe symptoms like low mood and irritability that do not significantly impair daily functioning) [19, 20]. Parent–child relationships as a core environmental factor in child psychological development, are widely recognized as a critical predictor of depression [14]. Previous studies have demonstrated that impaired family functioning, such as heightened parent–child conflict and low family closeness, could be detrimental to children’ depressive disorder (e.g., Qu et al., 2022). While the parent–child relationship is a multifaceted construct that can be defined in various ways depending on the theoretical framework, the present study focuses on two central dimensions: parent–child conflict and closeness. Closeness is manifested in emotional support, trust, and effective communication, while conflict is manifested in oppositional behavior or emotional detachment [21]. Attachment theory proposes that secure parent–child attachments reduce depression risk by fostering positive self-representations and adaptive emotion regulation strategies, thereby establishing constructive internal working models [22]. Conversely, insecure attachments significantly increase depression vulnerability through the consolidation of negative cognitive biases [20, 23]. Consequently, emotional detachment from parents and insecure attachment bonds may constitute a principal etiology of childhood depressive symptoms [15]. Longitudinal evidence indicates that parent–child conflict indirectly predicts depression via heightened neuroticism [24], while relational closeness reduces depression risk through enhanced emotional resilience [25]. The interpersonal theory of stress posits that parent–child relationship quality influences depression risk by shaping children’s social-behavioral functioning and emotion regulation capacity [26]. This framework suggests that strained parent–child relationships impair social adaptation and deplete emotional regulatory resources, exacerbating depression susceptibility under stress [27]. For instance, Li et al.’s [28] four-wave longitudinal study demonstrated positive associations between declining parent–child relationship quality and adolescent depression levels, with relational instability further predicting depression onset through increased cognitive load and emotional dysregulation. Additionally, the stress-buffering hypothesis specifies that robust social support systems mitigate the adverse mental health impacts of stressors by modulating cognitive appraisals, emotional reactivity, and coping strategies [29]. Existing research has validated that close parent–child relationships buffer against external stressors’ negative effects on child mental health [30, 31]. Despite extensive empirical investigations into parent–child relationships and childhood depression, substantial heterogeneity persists in reported effect magnitudes. Observed correlation coefficients range from − 0.54 [17] to − 0.09 [16], underscoring the necessity for systematic data integration to elucidate this association.

## Impact of moderator variables

Although parent–child relationships demonstrate robust associations with childhood depression, this linkage exhibits significant variability contingent upon moderating factors. These moderating influences may include child age, gender of both parent and child, and cultural background.

Child age constitutes a critical moderator in the parent–child relationship-depression association. During early childhood, when cognitive and emotion regulation capacities remain underdeveloped and parental dependency peaks, emotional support and attachment security in parent–child interactions may exert stronger predictive effects on depression [32]. Negative interactions at this developmental stage potentially exacerbate depression risk through reinforced negative self-schemas [33, 34]. Longitudinal evidence indicates that maternal disengagement during conflict tasks predicts heightened depressive symptoms in later childhood [35]. However, reduced autonomy demands and relatively manageable conflict frequency/intensity in childhood result in weaker direct effects compared to adolescence [36]. During adolescence (13–22 years), prefrontal cortex immaturity limits emotion regulation capacity while autonomy demands and social network diversification intensify [37, 38]. This neurodevelopmental context amplifies positive associations between parent–child conflict frequency and depression [39, 40]. Maternal “emotional reciprocity” toward adolescent anger expression particularly elevates depression risk in females [32, 36], while the buffering effects of emotional support attenuate. State space grid analyses by Zhang et al. [38] further reveal that highly predictable parent-adolescent conflict patterns strengthen positive associations between conflict intensity and depressive symptoms. Empirical syntheses confirm heightened sensitivity to parent–child relational influences during adolescence, attributable to psychosocial developmental characteristics [32, 35, 38]. Accordingly, this study aims to systematically investigate whether child age moderates the association between parent–child relationship and child depression.

The gender of both parents and children may significantly moderate the relationship between parent–child relationship and child depression. From the perspective of coping resources, males typically exhibit superior psychological resilience and self-efficacy, enabling them to better buffer the impact of stressful parent–child relationships on mental health [41]. In contrast, females tend to demonstrate greater vulnerability to depression due to their tendency to internalize negative emotions [42, 43]. For instance, parental conflict has been shown to have a stronger predictive effect on depressive symptoms in early adolescent girls [44]. A cohort study conducted in China further revealed that father-child conflict is only associated with depression in girls, while a close father-daughter relationship appears to have a more pronounced buffering effect [45, 46]. Moreover, the different functional roles that parents play in the parent–child relationship may have varying impacts on child depression [28, 47]. The differential functional roles of fathers and mothers in parent–child relationships may engender disparate influences on childhood depression [48]. Fathers are often described as forgotten contributors, exerting irreplaceable developmental influences through unique relational patterns [49]. Evidence suggests that paternal discipline and support primarily predict externalizing behaviors, while maternal care and emotional regulation more significantly impact internalizing symptoms such as depression [50]. Compared to paternal influences, maternal interpersonal sensitivity interacts more strongly with the quality of parent–child relationships in predicting child depression [51]. Overall, the association between parent–child relationships and depression in children may vary based on gender.

The association between parent–child relationships and depression demonstrates cross-cultural heterogeneity, with cultural contexts potentially moderating underlying mechanisms through their shaping of family values and emotional expression norms. Cross-cultural research indicates stronger effects of parent–child relationships on depression in collectivist cultures [52, 53]. Specifically, in collectivist contexts (e.g., China), Confucian filial piety cultures emphasizing familial harmony and parental authority render father-child relationships central to familial authority [54], where parent–child conflicts are often masked through emotional suppression and manifest in more covert forms [55]. In contrast, individualistic cultures (e.g., Western societies) exhibit more direct emotional confrontations in parent–child conflicts [56, 57], with adolescents’ heightened autonomy demands increasing sensitivity to communication breakdowns [24]. This cultural divergence further manifests through stronger buffering effects of parent–child closeness in collectivist contexts versus more pronounced negative impacts of conflict in individualistic settings [58]. Such differences may originate from collectivist societies’ embedding of parent–child relationships within familial honor systems, where conflict threatens group cohesion, versus individualistic societies’ prioritization of personal emotional authenticity [56, 59]. Collectively, cultural background serves as a critical moderator of parent–child relationship-depression associations. Consequently, this study examines the moderating role of cultural contexts across sampled populations.

Finally, this study will examine the influence of publication status, measurement instruments for childhood depression, and study design on the association between parent–child relationships and childhood depression. Additionally, the results of published studies will be compared with those from unpublished master’s theses to assess the consistency of conclusions. The investigation will further explore potential discrepancies in findings between cross-sectional and longitudinal studies within research examining parent–child relationships.

## The present study

Although attachment theory, family systems theory, and social learning theory each emphasize the role of parent–child interaction patterns in children’s psychological development from distinct perspectives, existing empirical studies remain inconsistent. On one hand, numerous investigations have explored the relationship between specific dimensions of parent–child relationships (e.g., parent–child closeness and conflict) and childhood depression, yet the magnitude of their association remains inconclusive. On the other hand, substantial heterogeneity in sample characteristics, cultural contexts, and study designs across current research necessitates systematic examination of the stability of parent–child relationship-depression associations under varying conditions.

As noted, empirical studies examining the parent–child relationship-childhood depression link have yielded inconsistent findings, with no existing meta-analysis to explain these discrepancies. To address this gap, this study employs a meta-analytic approach to synthesize previous research findings on the association between parent–child relationships and childhood depression, thereby enhancing conceptual understanding. The meta-analysis aims to resolve two core research questions: (1) quantifying the effect size of associations between parent–child relationships (encompassing two key dimensions: closeness and conflict) and childhood depression; and (2) investigating moderating effects of child age, cultural context, and study design on these associations.

## Methods

### Study design

The present meta-analysis was rigorously conducted in adherence to the Preferred Reporting Items for Systematic Reviews and Meta-Analyses (PRISMA 2020) guidelines, an evidence-based framework designed to enhance methodological transparency, reproducibility, and reporting quality in systematic evidence synthesis. To ensure transparency and avoid unintentional duplication of effort, the protocol for this meta-analysis was registered in the International Prospective Register for Systematic Reviews (PROSPERO 2025 CRD420251028530).

### Search strategy

In accordance with PRISMA guidelines, a comprehensive search strategy was implemented across major academic databases. The search encompassed Web of Science, EBSCO, ProQuest, PubMed, PsycINFO, and China National Knowledge Infrastructure (CNKI), utilizing carefully designed search terms to identify relevant studies: (1) Parent (e.g., parent* OR parental* OR guardian* OR caregiver* OR father* OR dad* OR mother* OR mom* OR family); (2) Child (e.g., child* OR infant* OR baby OR babies* OR toddler* OR preschool* OR kid* OR youth* OR teen* OR adolescent* OR young*); and (3) Parent–child relationship (e.g., “parent–child relationship*” OR “father-child relationship*” OR “mother–child relationship*” OR “child-mother relationship*” OR “child-father relationship*” OR “paternal-child relationship*” OR “maternal-child relationship*” OR “parent–child interaction*” OR “mother–child interaction*” OR “father-child interaction*” OR “dyadic communication*” OR “parent–child communication*” OR “mother–child communication*” OR “father-child communication*” OR Reciprocity* OR Synchrony* OR “Dyadic Mutuality*” OR Mutuality* OR “emotional availability*” OR “attachment relationships*” OR “father-child attachment relationships*” OR “mother–child attachment relationships*”).

To mitigate potential measurement bias, search terms explicitly referencing children’s depressive symptoms or depression diagnoses were excluded from the search strategy, following methodological recommendations by Frandsen et al. [60]. The final database search was completed in October 2024. Furthermore, manual searches of reference lists from included studies and seminal reviews in the field of parent–child relationship dynamics and childhood affective disorders were conducted. This citation tracking procedure persisted until theoretical saturation was achieved, with three consecutive screening rounds yielding no new eligible studies.

### Inclusion and exclusion criteria

The inclusion criteria for the literature in this meta-analysis were as follows: (1) Studies involving children and adolescents under the age of 22 years, with a mean sample age of ≤ 18 years to ensure developmental appropriateness; (2) Investigations examining the association between parent–child relationship dynamics (e.g., attachment security, parent–child conflict, emotional availability, or communication quality) and children’s depressive symptoms or clinically diagnosed depression; (3) Use of validated instruments to assess both parent–child relationship characteristics (e.g., Parental Bonding Instrument, Child-Parent Relationship Scale) and depression outcomes (e.g., Children’s Depression Inventory [CDI], Center for Epidemiologic Studies Depression Scale [CES-D]); (4) Clear reporting of correlation coefficients (*r*), standardized regression coefficients (*β*), odds ratios (OR), or provision of inferential statistics (*t*-values, *F*-values, χ^2^) that can be converted to effect sizes; (5) Reporting of sample size and demographic characteristics; (6) Inclusion of cross-sectional, longitudinal, or intervention study designs; and (7) Availability of studies published in peer-reviewed journals in English or Chinese.

Conversely, studies were excluded if they (1) focused on mental health outcomes other than depression (e.g., emotional dysregulation, externalizing behaviors); (2) had a sample size of < 30 to ensure adequate statistical power; (3) were non-empirical reports (e.g., case studies, reviews, theoretical papers); or (4) examined parental psychopathology as the primary predictor rather than relational processes.

### Selection procedure

Articles were reviewed by at least two independent reviewers until consensus was reached on the inclusion or exclusion of an article. The study selection process adhered to PRISMA guidelines through a three-stage deduplication protocol ensuring methodological rigor. First, duplicate records were systematically removed using database-native functions, followed by iterative manual cross-checking of metadata fields (e.g., DOI, author-year-title composites), culminating in consensus confirmation by independent researchers. Each publication underwent a dual-phase evaluation: initial triage based on title/abstract relevance to parent–child relational predictors and childhood depression, followed by rigorous full-text appraisal against predefined inclusion criteria. To minimize measurement bias inherent in relational studies, a double-blind procedure was implemented where raters remained unaware of each other’s decisions during screening. Any discrepancies between the raters were resolved through discussion with the corresponding author until a consensus was reached.

### Data extraction

The following data were extracted: (1) first author names and publication year, (2) correlation coefficient, (3) the number of study samples, (4) gender distribution of children (measured by boy ratio), (5) average age of children, (6) measurement instrument, (7) parent–child relationship group (father-child relationship vs. mother–child relationship), (8) participant’s country, and (9) publication type (journal paper vs. Dissertation).

In the process of data extraction, we adhered to established meta-analytic protocols for developmental studies. First, effect sizes were calculated using independent samples, with each unique dyad (parent–child pair) contributing one effect size for a specific parent–child relational dimension (e.g., attachment security, conflict frequency) and children’s depression outcome. Second, when multiple effect sizes from the same cohort could be classified into distinct moderator categories (e.g., maternal vs. paternal relationship quality, depressive symptoms vs. clinical diagnosis), they were treated as separate entries for subgroup analyses. For instance, studies reporting differential effects of father-child conflict and mother–child conflict on adolescent depression were coded independently. Third, to preserve effect size independence, when multiple measurements of the same relational construct (e.g., repeated assessments of parent–child conflict) predicted depression outcomes within a single study, we employed multilevel modeling rather than simple averaging, per contemporary psychometric standards. Fourth, for longitudinal designs, only the temporal association between baseline parent–child relationship quality and endpoint depression severity was retained, controlling for autoregressive effects. Finally, developmentally significant moderators (e.g., child gender, age) reported in stratified analyses were systematically coded for heterogeneity examination.

To ensure coding reliability, two researchers independently coded the data, and consistency was assessed. Intraclass correlation coefficients (ICC) were used for continuous variables, and Cohen’s kappa (κ) was used for categorical variables. Any discrepancies were resolved through discussion and consensus.

### Risk of bias assessment

The risk of bias for all included studies was assessed using the Quality Assessment Tool for Observational Cohort and Cross-Sectional Studies [61]. This assessment tool consists of 14 items, with each item providing five response options: yes, no, cannot be determined, not reported, and not applicable. A score of 1 was allocated for the response “yes,” whereas the other options were not assigned any points. The cumulative score was utilized to classify the quality of the literature into three categories: good (total score > 7), fair (total score ranging from 5 to 7), and poor (total score < 5). High-quality studies are typically distinguished by their low risk of bias. The coding procedure was conducted independently by two authors, and their agreement on the total score was found to be highly consistent (ICC = 0.986). Any disagreements that arose during the coding were resolved through consensus discussions.

### Statistical analyses

The meta-analysis was conducted using R (version 4.4.3-mac; R Foundation for Statistical Computing) with the meta and meta for packages. Effect sizes were measured using correlation coefficients (*r*). Before the meta-analysis, all correlation coefficients were transformed into Fisher *z* scores. After the analysis, Fisher *z* values were converted back to Pearson correlation coefficients for easier interpretation. Given the anticipated heterogeneity in measurement methods for parent–child relationships and children’s depression, as well as variations in participant characteristics (eg, gender, age, cultural background) across the included studies, we used a random effects model for our meta-analysis. Heterogeneity was assessed using the *Q* and* I*^2^ test statistics. A significant *Q* test or an *I*^2^ value above 75% indicated substantial heterogeneity, supporting the use of a random effects model [62].

For the moderator analysis, continuous moderators were analyzed using meta-regression, while categorical moderators were analyzed using subgroup analysis. The significance of moderators was assessed using the *Q* statistic. As recommended by Huang [63], each subgroup should include a minimum of 3 studies for the analysis of categorized moderating variables.

Publication bias refers to the tendency for significant results to be more likely to be published, while nonsignificant results may remain unpublished. To address publication bias, this study included both published journal papers and unpublished theses when selecting literature, which helped to control the influence of publication bias on the research findings. Additionally, to ensure the reliability of the meta-analysis results, funnel plot and Egger regression intercept were used to assess the presence of publication bias. If the funnel plot exhibited a symmetrical inverted funnel shape and the Egger regression intercept was nonsignificant, publication bias was considered negligible.

## Results

### Search results

**Fig. 1:**
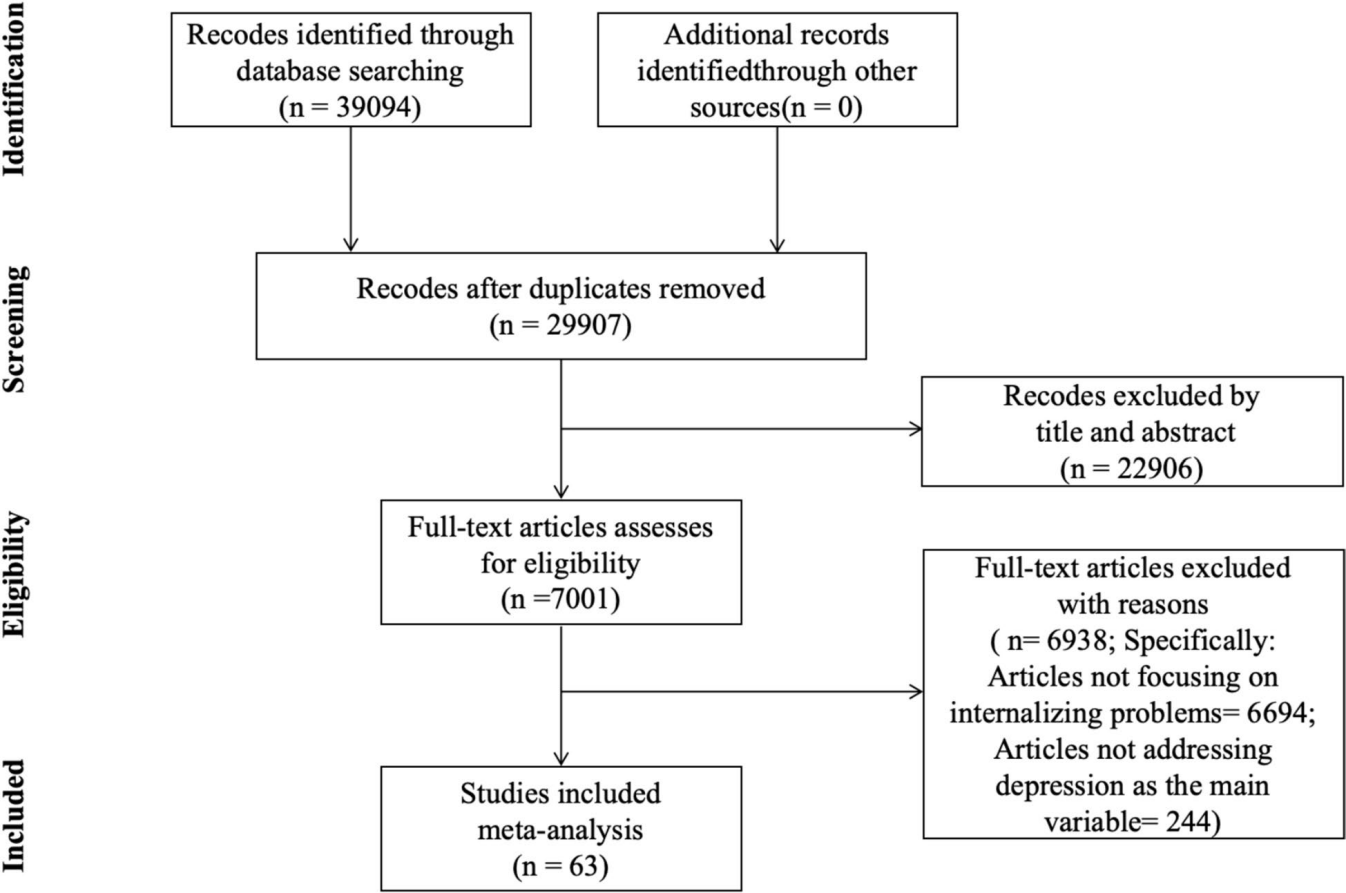
PRISMA Flow Diagram and Study Selection Process

This study adhered to the Preferred Reporting Items for Systematic Reviews and Meta-Analyses (PRISMA) guidelines. Through keyword searches in databases including PsycInfo, PubMed, and CNKI, 39,094 articles were initially identified, with 29,907 remaining after duplicate removal. Based on predefined inclusion and exclusion criteria, 22,906 studies were excluded through title and abstract screening. Full-text assessments were conducted for the remaining 7001 studies, resulting in the exclusion of 6938 studies that did not measure depression as an outcome variable. Ultimately, 63 studies met all inclusion criteria and were included in the meta-analysis. The detailed screening process is illustrated in Fig. 1.

### Study characteristics

This meta-analysis incorporated 97 effect sizes from 63 independent studies, with a cumulative sample of 70,687 participants (Table 1). Individual study sample sizes ranged from 46 to 11,321, with a mean participant age of 13.33 years (SD 1.12) and an age range of 5.42–19.85 years. Female participants accounted for 50.55% of the total sample. The included studies spanned publication years from 2001 to 2024, with the majority (*k* = 23) published in 2021 or later. Geographically, 36 studies originated from Asia (China and Korea), while 27 were conducted in Europe and North America (United States, Canada, and France). Methodologically, 41 studies (65.08%) employed cross-sectional designs, compared to 22 longitudinal studies (34.92%). The Literature comprised 40 peer-reviewed journal articles and 23 unpublished master’s theses. Parent–child relationships were predominantly assessed through standardized questionnaires, including the Parent–Child Relationship Scale (CPRS; *k* = 9), Network of Relationships Inventory, Conflict Behavior Questionnaire (CBQ), and Parental Environment Questionnaire (PEQ). Depression measurements primarily utilized established instruments: the Children’s Depression Inventory (CDI; k = 14), Center for Epidemiological Studies Depression Scale (CES-D), Beck Depression Inventory (BDI), and Self-Rating Depression Scale (SDS). Two studies employed the Symptom Checklist-90 (SCL-90) as a multidimensional mental health assessment. Quality appraisal classified 5 studies as moderate Quality and 58 as good quality.

**Table 1:** Characteristics of the 63 studies included in the analysis

Authors (publication year)	Participants, n	Age (y), mean (SD)	Gender (boys), %	Parent–child relationship type	Parent–child relationship results	Publication type	Research design	Country	Literature quality	Measurement tool	
										Parent–child relationship	Depression
Arria et al. [80]	1249	N	0.52	M & F	Conflict	Journal	Longitudinal research	America	Good	QRI	BDI
Bradford et al. [81]	641	15.10 (1.57)	0.48	P	Conflict	Journal	Cross-sectional research	America	Good	N	CDI
Briere et al. [82]	3864	N	0.46	P	Conflict	Journal	Longitudinal research	Canada	Good	MASPAQ	CES-D
Cao. [83]	498	N	0.52	P	Conflict & Closeness	Dissertation	Cross-sectional research	China	Good	PCRS-C	CES-D
Chiang and Bai. [55]	2292	12.00	0.51	P	Conflict & Closeness	Journal	Longitudinal research	China	Good	PCCS3	SCL-90
Choi et al. [84]	656	12.97 (1.00)	–	M	Conflict	Journal	Longitudinal research	Korea	Good	CBQ	CDI
Choi et al. [85]	784	15.00 (1.91)	0.48	P	Conflict	Journal	Longitudinal research	America	Good	PARQ	CDI
Criss and Shaw. [86]	122	N	1.00	M	Conflict	Journal	Longitudinal research	America	Good	STRS	CDI
Cummings et al. [87]	295	13.10 (0.53)	0.49	M & F	Conflict	Journal	Longitudinal research	America	Good	SCIFF	CES-D
Dai. [88]	1023	10.27 (0.78)	0.55	P	Conflict	Dissertation	Longitudinal research	China	Good	PEQ	CES-D
Deng et al. [89]	1561	N	0.54	P	Conflict & Closeness	Journal	Cross-sectional research	China	Good	PIS、PACS	SDS
Dong et al. [90]	7010	16.19 (0.73)	0.44	P	Conflict	Journal	Cross-sectional research	China	Good	PBI	PHQ-9
Du. [91]	2279	N	0.51	M & F	Closeness	Dissertation	Cross-sectional research	China	Good	N	MMHI-60
Ehrlich et al. [92]	189	16.50	0.38	M & F	Conflict	Journal	Cross-sectional research	America	Good	TCC	CDI
Galan et al. [93]	252	11.98 (1.19)	0.44	P	Conflict	Journal	Longitudinal research	America	Good	ALEXSA	ALEXSA
Gao. [94]	450	5.42 (0.47)	0.52	M & F	Conflict	Dissertation	Cross-sectional research	China	Good	CPRS	PFC-S
Ge et al. [95]	756	12.82 (1.95)	N	M & F	Closeness	Journal	Longitudinal research	America	Good	PCRSH	CDI
Guo. [66]	620	12.41 (0.61)	0.56	M	Closeness	Dissertation	Longitudinal research	China	Good	Self	CES-D
Hou et al. [96]	350	17.04 (0.73)	0.42	M & F	Conflict	Journal	Longitudinal research	America	Good	AAFCS	CES-D
Howard et al. [97]	322	14.60 (1.50)	0.45	M & F	Conflict	Dissertation	Longitudinal research	America	Good	CBQ	CDRS-R
Huang. [98]	346	13.87 (0.74)	0.50	M	Conflict	Dissertation	Cross-sectional research	China	Good	FES	CDI
Kim. [99]	11,321	N	0.48	N	Conflict	Journal	Longitudinal research	America	Good	N	N
Lamis and Jahn. [100]	552	19.85 (1.66)	0.23	P	Conflict	Dissertation	Cross-sectional research	America	Good	PEQ	BDI
Leung. [101]	1570	12.61 (0.76)	0.51	M & F	Conflict	Journal	Longitudinal research	China	Good	PAC	HADS
Leung. [101]	1735	12.63 (0.79)	0.53	M & F	Conflict	Journal	Cross-sectional research	China	Good	PAC	HADS
Li. [51]	751	N	N	P	Conflict	Dissertation	Cross-sectional research	China	Good	Self	CDI
Li. [102]	1055	14.62 (0.79)	0.53	M & F	Conflict	Dissertation	Cross-sectional research	China	Fair	PCCCS	CES-D
Lim et al. [103]	81	N	0.57	M	Conflict	Journal	Cross-sectional research	America	Good	CAM	GCS
Liu et al. [46]	438	13.80 (0.52)	0.56	M & F	Conflict	Journal	Cross-sectional research	China	Good	NRI	CDI
Liu et al. [104]	1594	13.13 (1.54)	0.49	P	Conflict	Journal	Cross-sectional research	China	Good	PEQ	CES-D
Liu. [105]	480	13.77 (2.37)	0.54	P	Conflict	Dissertation	Cross-sectional research	China	Good	NRI	CDI
Miao. [106]	1934	13.20 (0.35)	0.51	M & F	Conflict	Dissertation	Longitudinal research	China	Good	CBQ、FACES	CBCL
Nathans et al. [107]	242	N	N	P	Closeness	Dissertation	Cross-sectional research	America	Good	NLSY-SF	CES-D
Nowakowski-Sims and Rowe. [108]	80	15.00 (1.55)	0.52	P	Conflict & Closeness	Dissertation	Cross-sectional research	America	Good	IPPA-R、CAS	BDI
Paul. [109]	46	8.64 (2.15)	0.43	M & F	Closeness	Journal	Cross-sectional research	France	Good	SAGA	TSCC
Peng et al. [48]	654	12.94 (1.11)	0.56	F	Conflict	Dissertation	Cross-sectional research	China	Fair	CBS	CES-D
Pu. [110]	1980	N	0.53	P	Conflict	Dissertation	Cross-sectional research	China	Good	CFPS	CES-D
Qu et al. [111]	879	13.14 (1.31)	0.49	P	Conflict & Closeness	Journal	Longitudinal research	China	Good	NRI	DSM–5
Roblyer et al. [112]	91	N	0.55	P	Conflict	Journal	Cross-sectional research	America	Good	PCDC	MFQ
Russell et al. [113]	271	N	N	P	Closeness	Dissertation	Longitudinal research	America	Good	CPRS	MDI
Sentse and Laird. [114]	218	N	0.49	M	Conflict	Dissertation	Longitudinal research	America	Good	PBI、CRPBIS	MDS
Shen. [6]	154	N	0.50	P	Conflict	Journal	Cross-sectional research	China	Fair	PCRT	SCL-90
Sichko et al. [115]	106	10.25 (1.09)	0.51	M	Closeness	Journal	Cross-sectional research	America	Good	IOS	CDI
Smith et al. [35]	601	N	0.49	M & F	Conflict	Journal	Longitudinal research	America	Good	CPRS	CDI
Stein et al. [116]	171	14.02	0.47	P	Conflict	Journal	Cross-sectional research	America	Good	NRI	ACSQ
Strickland et al. [117]	1960	N	0.44	P	Closeness	Journal	Cross-sectional research	America	Good	AAH-PCS	SA-45
Venkatesh et al. [118]	401	14.12 (2.12)	0.48	P	Closeness	Journal	Cross-sectional research	China	Good	IPPA	DP6
Wang et al. [119]	2041	14.11 (2.42)	0.47	M & F	Conflict	Journal	Cross-sectional research	China	Good	PCQ	SDS
Wang. [120]	715	N	N	M & F	Conflict & Closeness	Dissertation	Cross-sectional research	China	Good	CBQ、FACES	CES-D
Wang. [121]	960	14.86 (1.64)	0.49	M & F	Closeness	Dissertation	Cross-sectional research	China	Good	RFCPI	CES-D
Withers et al. [122]	498	13.34	0.53	N	Conflict & Closeness	Journal	Cross-sectional research	America	Good	HOME	N
Wu and Zhang. [123]	349	N	0.52	P	Conflict	Journal	Cross-sectional research	China	Good	PCRT	SCL-90
Xi and Wang. [124]	551	N	0.52	P	Conflict	Journal	Cross-sectional research	China	Good	CPRS	DASS
Xiao et al. [125]	452	14. 18 (0. 95)	0.44	M & F	Conflict & Closeness	Journal	Cross-sectional research	China	Good	CPRS	CDI
Yan et al. [45]	685	N	0.50	M & F	Conflict & Closeness	Dissertation	Longitudinal research	America	Good	CPRS	CDI
Yang and Wu. [126]	949	13.00 (0.89)	0.00	p	Conflict	Journal	Cross-sectional research	China	Fair	PCCS-FD	CBCL
Yang et al. [127]	852	14. 30 (0. 48)	0.50	M	Conflict & Closeness	Journal	Cross-sectional research	China	Good	CPRS、FACES II	CDI
Yang et al. [20]	797	N	0.52	P	Conflict	Journal	Cross-sectional research	China	Good	PC-CMUS	CDI
Yang. [128]	1708	14.30 (0.48)	0.50	M	Conflict & Closeness	Dissertation	Cross-sectional research	China	Fair	MCI-M、FACES -II	CDI
Yeh et al. [129]	603	16.95 (0.78)	0.38	M & F	Conflict	Dissertation	Cross-sectional research	China	Good	PIS-SF	KMHQ
Yu. [130]	2844	9.86 (0.35)	N	P	Conflict	Journal	Longitudinal research	Korea	Good	PCC5	CSDM
Zeiders et al. [16]	738	10.4 (0.55)	0.58	P	Conflict	Journal	Cross-sectional research	America	Good	PACS	CES-D
Zhou et al. [17]	1021	10.33 (0.98)	0.56	M	Conflict & Closeness	Journal	Cross-sectional research	China	Good	CPRS	N

N, Not reported; M, Mother–child relationship; F, Father-child relationship; P, Parent–child relationship; QRI, Quality of Relationship Inventory; MASPAQ, Mesures de l’adaptation sociale et personnelle pour les adolescents Québécois; Self, Self-designed scale; PCCS3, Parent–Child Conflict Scale (3-item); CBQ, Conflict Behavior Questionnaire; PARQ, Parent-Adolescent Relationship Questionnaire; STRS, Student–Teacher Relationship Scale; SCIFF, The System for Coding Interactions and Family Functioning; PEQ, Parental Environment Questionnaire; PCCS-conflict, Parent–child Conflict Scale; PCCS-cohesion, Parent–child Cohesion Scale; PBI, The Parental Bonding Instrument; TCC, Topics of Conflict Checklist; ALEXSA, Assessment of Liability and Exposure to Substance use and Antisocial Behavior; CPRS, Parent–Child Relationship Scale; PCRSH, Parent–Child Relationship Scale; AAFCS, Asian American Family Conflicts Scale; FES, Family Environment Scale; FACS, Father-Adolescent Conflict Scale; MACS, Mother-Adolescent Conflict Scale; Self, Self-designed scale; PCCCS, Parent–child conflict content scale; CAM, Child’s Attitude to Mother scale; NRI, Network of Relationships Inventory; CBQ, The Conflict Behavior Questionnaire; FACES, Family Adaption and Cohesion Evaluation Scales; NLSY-SF, National Longitudinal Survey of Youth-Short Form; IPPA-R, Inventory of Parent and Peer Attachment; SAGA, Systemic Analysis of Group Affiliation; CBS, Conflict Behavior Scale; CFPS, China Family Panel Studies; PCDC, Parent–Child Difficulties Checklist; PBI, Parental Behavior Inventory; PCRT, Parent–Child Relationship Test; IOS, Inclusion of Others in the Self Scale; AAH-PCS, Adapted Add Health Wave II Parental Closeness Scale; IPPA, Inventory of Parent and Peer Attachment; PCQ, Parent–child Conflict Questionnaire; RFCPI, the revised family communication pattern instrument; HOME, The Home Observation for Measurement of the Environment; PACQ, Parent-adolescent conflict questionnaire; CPRS, Parent–Child Relationship Scale; FACES II, Family Adaptability and Cohesion Evaluation Scales II; PACS-R, Parent-Adolescent Conflict Scale; FACES -II, Family Adaptability and Cohesion Evaluation Scales II; PIS-SF, Parent–Child Interaction Scale-Short Form; PCC5, Parent–Child Conflict Scale (five items); PACS, Parent–Adolescent Conflict Scale; BDI, The Beck Depression Inventory; CDI, Children’s Depression Inventory; CES-D, Center for Epidemiological Studies Depression Scale; SCL-90R, The Chinese Version of the Symptom Checklist-90-Revised; CDIA, Angold’s Children’s Depression Inventory; SDS, Self-Rating Depression Scale; PHQ-9, Patient Health Questionnaire-9; MMHI-60, Mental Health Inventory for Middle School Students; ALEXSA, The Assessment of Liability and Exposure to Substance use and Antisocial behavior; PFC-S, Preschool Feelings Checklist-Screener; CDRS-R, Children’s Depression Rating Scale-Revised; BDI-II, The Beck Depression Inventory–II; HADS, Hospital Anxiety and Depression Scale; GCS, Generalized Contentment Scale; CBCL, Child Behavior Checklist; TSCC, Trauma Symptom Checklist for Children; DSM–5, Diagnostic and Statistical Manual of Mental Disorders; MFQ, Mood and Feelings Questionnaire; MDI, Major Depression Inventory; MDS, Modified Depression Scale; SCL-90, Symptom Checklist-90; ACSQ, Adolescent Cognitive Style Questionnaire; SA-45, Symptom Assessment 45 Questionnaire; DP6, Depression (six items); DASS, Depression Anxiety Stress Scale; CDI-S, Short Version of Children’s Depression Inventory; KMHQ, Ko’s Mental Health Questionnaire

### Heterogeneity tests

The heterogeneity tests revealed significant variation across studies: for parent–child conflict and child depression, *Q* = 1353.32 (df = 66, *P* < 0.001); for parent–child closeness and child depression, *Q* = 626.42 (df = 29, *P* < 0.001), indicating substantial heterogeneity among effect sizes included in the meta-analysis (see Table 2). *I*^2^ index analysis demonstrated exceptionally high heterogeneity, with values of 95.1% for parent–child conflict and 95.4% for parent–child closeness in relation to child depression, both substantially exceeding the 75% threshold for high heterogeneity. Notably, over 95% of the variability in effect sizes originated from true between-study differences rather than sampling error. These findings suggest that observed discrepancies in effect sizes may be attributable to specific study characteristics, justifying the use of a random-effects model for analysis and necessitating further investigation of potential moderator variables.

**Table 2:** Results of Effect Size Heterogeneity Test

	Heterogeneity						Tau^2^	
	*k*	*Q*	*df (Q)*	*P*	*I*^*2*^ (%)	Tau^2^	Variance	Tau
Parent–child conflict	67	1353.32	66	< 0.001	95.1	0.0145	< 0.001	0.1204
Parent–child closeness	30	626.42	29	< 0.001	95.4	0.0318	< 0.001	0.1784

### Overall relation between parent–child relationship and children’s depression

**Fig. 2:**
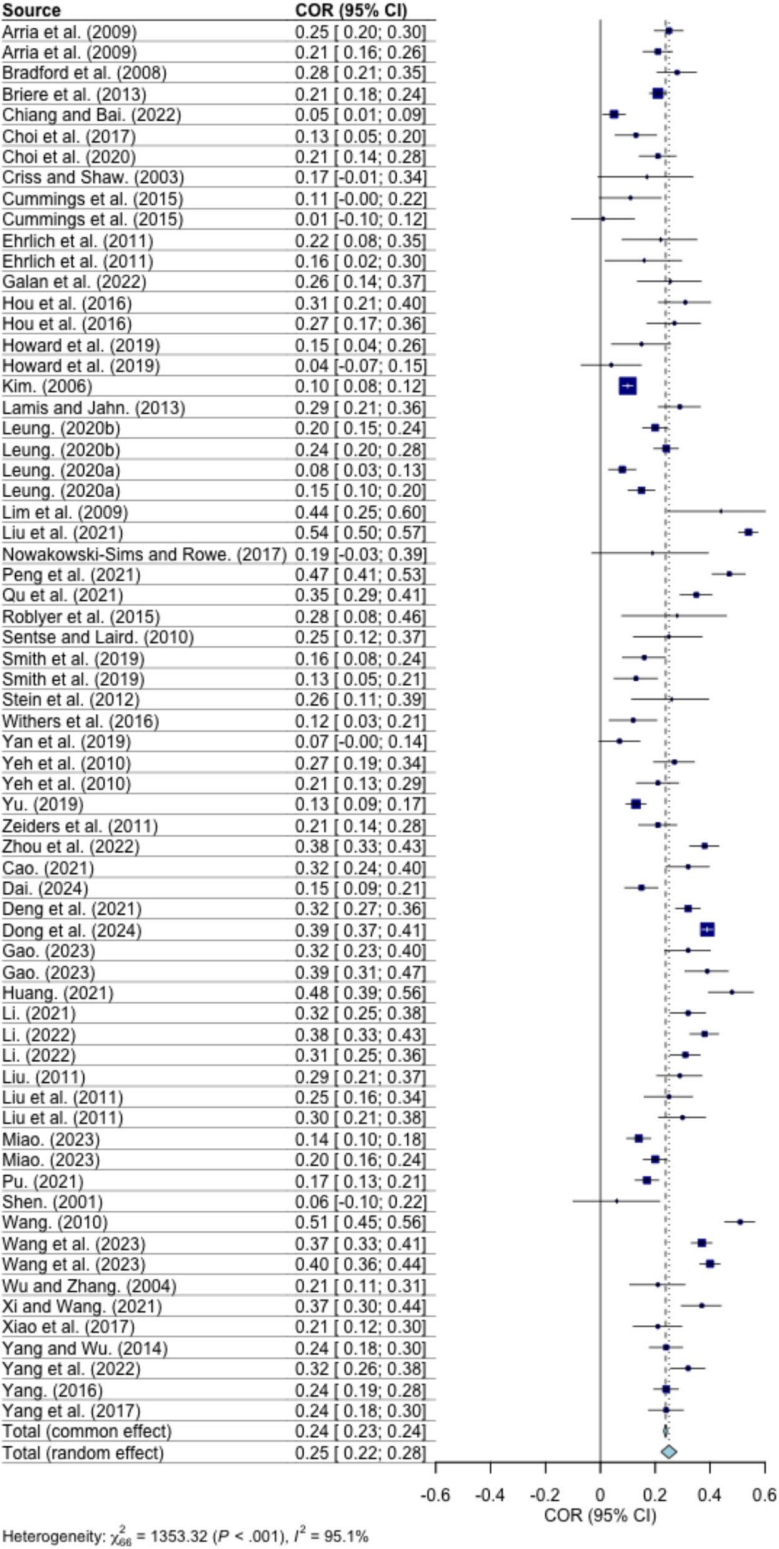
Forest plot for correlation between parent–child conflict and children’s depression

**Fig. 3:**
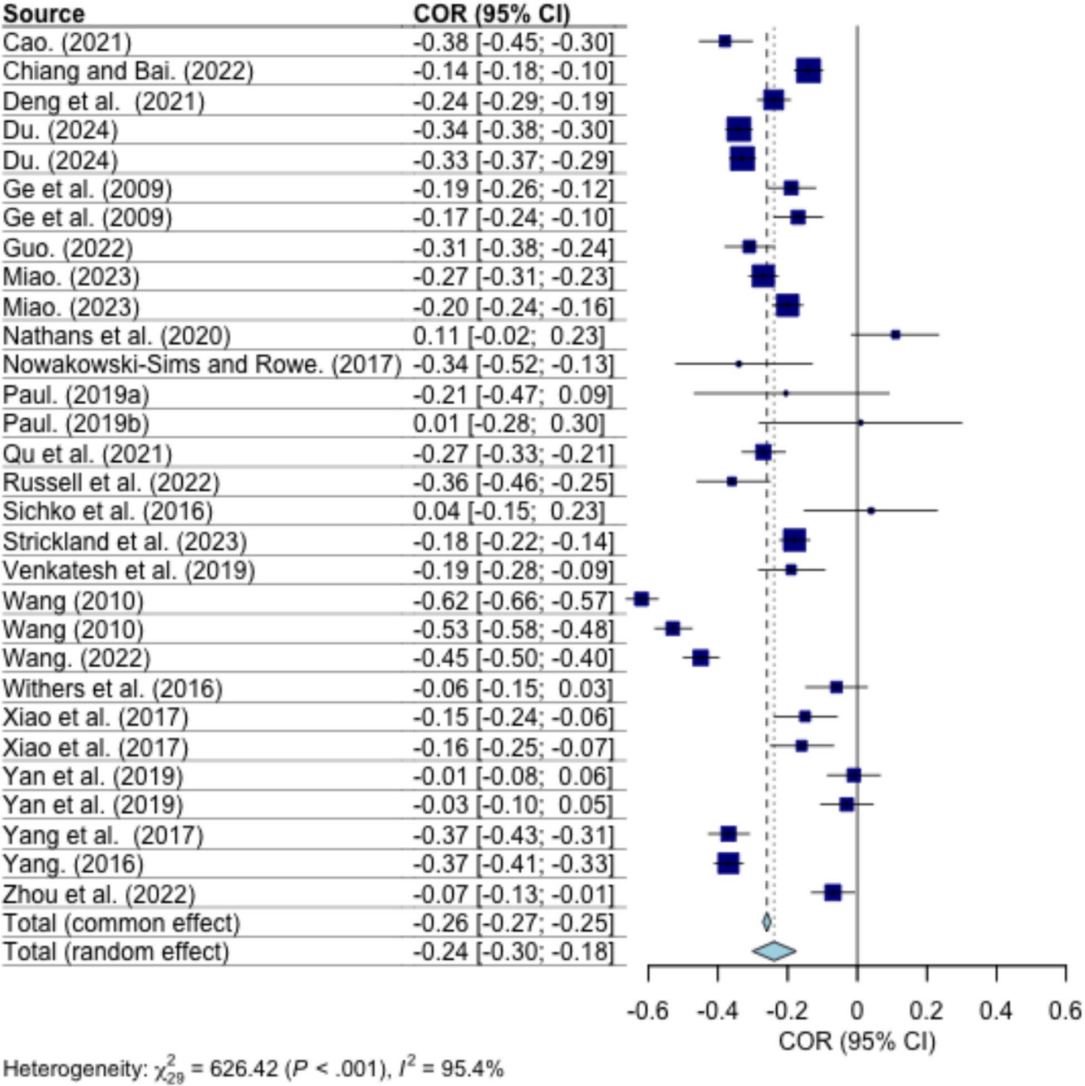
Forest plot for correlation between parent–child closeness and children’s depression

This study employed a random-effects model to examine the relationship between parent–child relationships and childhood depression. A total of 97 effect sizes were included in the analysis, comprising 67 effect sizes examining parent–child conflict and childhood depression, and 30 effect sizes examining parent–child closeness and childhood depression. The results revealed a significant positive correlation between childhood depression and parent–child conflict (*r* = 0.25; 95% CI = 0.22 to 0.28) and a significant negative correlation with parent–child closeness (*r* = − 0.24; 95% CI = − 0.18 to − 0.30), as illustrated in Figs. 2 and 3. Following Lipsey and Wilson’s classification criteria, correlation coefficients can be categorized as low (| *r* |≤ 0.1), moderate (0.1 <| *r* |< 0.4), or high (| *r* |≥ 0.4). In this study, the average effect sizes for the correlations between childhood depression and both parent–child conflict and closeness fell within the moderate range.

### Analysis of moderator variables

A moderator analysis was conducted for child age, country, and study design. Meta-regression results indicated that child age significantly moderated the association between parent–child closeness and child depression (*B* = − 0.048; 95% CI = − 0.02 to − 0.07; *z* = − 3.40; *P* = 0.001), but did not significantly moderate the relationship between parent–child conflict and child depression (*B* = 0.003; 95% CI = − 0.01–0.02; *z* = 0.379; *P* = 0.71). Furthermore, child gender did not significantly moderate the relationships between parent–child conflict (*B* = − 0.007; 95% CI = − 0.29–0.31; *z* = 0.042; *P* = 0.97) or parent–child closeness (*B* = − 0.515; 95% CI = − 2.20–1.17; *z* = − 0.598; *P* = 0.55) and child depression.

Cultural context may moderate the link between parent–child relationships and depression. Our meta-analysis found that most studies focused on China (collectivist) and the US (individualist), with respective sample sizes of 38 and 26. These two countries were selected as cultural representatives due to the sufficient number of studies. Other countries were excluded from moderation comparisons due to insufficient sample sizes (*n* < 3), though their findings are descriptively analyzed to offer a broader perspective. Specifically, in addition to the studies from China and the USA, our meta-analysis included four other studies: Briere et al. (2013) from Canada, Choi et al. (2017) and Yu (2019) from South Korea, and Paul (2019) from France. Furthermore, during the screening of depression measurement instruments, only scales used ≥ 5 times were retained to control for methodological heterogeneity. This resulted in the inclusion of two instruments: the CES-D (Center for Epidemiological Studies Depression Scale) and the CDI (Children’s Depression Inventory). Tables 3 and 4 present the results for the categorical moderators: (1) Cross-cultural moderation analysis revealed a significant moderating effect of cultural background on the association between parent–child conflict and child depression (*Q* = 19.20, *P* < 0.0001). The moderating effect was strongest in the Chinese sample (*r* = 0.29, 95% CI = 0.25 to 0.33), significantly higher than in the US sample (*r* = 0.15, 95% CI = 0.16 to 0.23). Study design also showed a significant moderating effect on the relationship between parent–child conflict and child depression (*Q* = 34.37, *P* < 0.0001), with larger effect sizes found in cross-sectional studies (*r* = 0.30, 95% CI = 0.27 to 0.34). However, publication status did not have a significant moderating effect (*Q* = 1.24, *P* = 0.27). (2) Cultural background had a significant moderating effect on the association between parent–child closeness and child depression (*Q* = 11.10, *P* = 0.004), with the largest effect size observed in the Chinese sample (*r* = − 0.31, 95% CI = − 0.38 to − 0.24). Similarly, publication status demonstrated a significant moderating effect (*Q* = 5.32, *P* = 0.02). However, study design did not significantly moderate the relationship between child depression and parent–child closeness (*Q* = 1.28, *P* = 0.26). (3) Parental role and depression measurement instrument failed to show significant moderation effects on the relationships between parent–child conflict (*Q* = 0.38, *P* = 0.83; *Q* = 2.27, *P* = 0.32) or parent–child closeness (*Q* = 0.21, *P* = 0.89; *Q* = 3.55, *P* = 0.17) and depression.

**Table 3:** Categorical moderator analysis of parent–child conflict and children’s depression

Moderator variables	Studies, n	*r*	95% CI	*Q* test (*df*)	*P* value
Types of parent–child conflict				0.38 (2)	0.83
Parent–child conflict	30	0.25	[0.21, 0.30]		
Father-child conflict	15	0.23	[0.17, 0.30]		
Mother–child conflict	22	0.26	[0.21, 0.30]		
Country				19.20 (2)	< 0.0001
America	26	0.15	[0.16, 0.23]		
China	38	0.29	[0.25, 0.33]		
Study design				34.37 (1)	< 0.0001
Cross-sectional	41	0.30	[0.27, 0.34]		
Longitudinal	26	0.17	[0.13, 0.20]		
Publication status				1.24 (1)	0.27
Journal	44	0.24	[0.20, 0.27]		
Dissertation	23	0.28	[0.22, 0.33]		
Depression Scale				2.27 (2)	0.32
CES-D	12	0.29	[0.19, 0.39]		
CDI	17	0.22	[0.19, 0.26]		
Other standardized scales	38	0.25	[0.21, 0.28]

**Table 4:** Categorical moderator analysis of parent–child closeness and children’s depression

Moderator variables	Studies, n	*r*	95% CI	*Q* test (*df*)	*P* value
Types of parent–child closeness				0.21 (2)	0.89
Parent–child closeness	10	− 0.22	[0.12, 0.32]		
Father-child closeness	7	− 0.27	[0.10, 0.42]		
Mother–child closeness	13	− 0.24	[0.14, 0.33]		
Country				11.10 (2)	0.004
America	10	− 0.12	[− 0.21, − 0.02]		
China	18	− 0.31	[− 0.38, − 0.24]		
Others	2	− 0.10	[− 0.33, 0.15]		
Study design				1.28 (1)	0.26
Cross-sectional	20	− 0.26	[− 0.34, − 0.17]		
Longitudinal	10	− 0.20	[− 0.26, − 0.13]		
Publication status				5.32 (1)	0.02
Journal	15	− 0.17	[− 0.22, − 0.12]		
Dissertation	15	− 0.31	[− 0.40, − 0.21]		
Depression Scale				3.55 (2)	0.17
CES-D	6	− 0.38	[− 0.56, − 0.17]		
CDI	9	− 0.22	[− 0.27, − 0.17]		
Other standardized scales	15	− 0.17	[− 0.26, − 0.07]		

### Publication bias and sensitivity analysis

The reliability and validity of meta-analytic findings depend on the absence of publication bias, particularly in the context of high between-study heterogeneity. This study employed multiple complementary methods to assess potential publication bias: visual inspection of funnel plots, Egger’s regression test, and Duval and Tweedie’s Trim-and-Fill analysis.

For the association between parent–child conflict and childhood depression, Egger’s regression test indicated no significant asymmetry (intercept = 1.28, *p* = 0.27). The Trim-and-Fill analysis identified only 3 potentially missing studies. After adjustment, the effect size (*r* = − 0.26, 95% CI = − 0.33 to − 0.19) showed minimal change from the original estimate (r = − 0.24), remaining highly significant (*p* < 0.001). This pattern suggests that publication bias has minimal impact on this association.

**Fig. 4:**
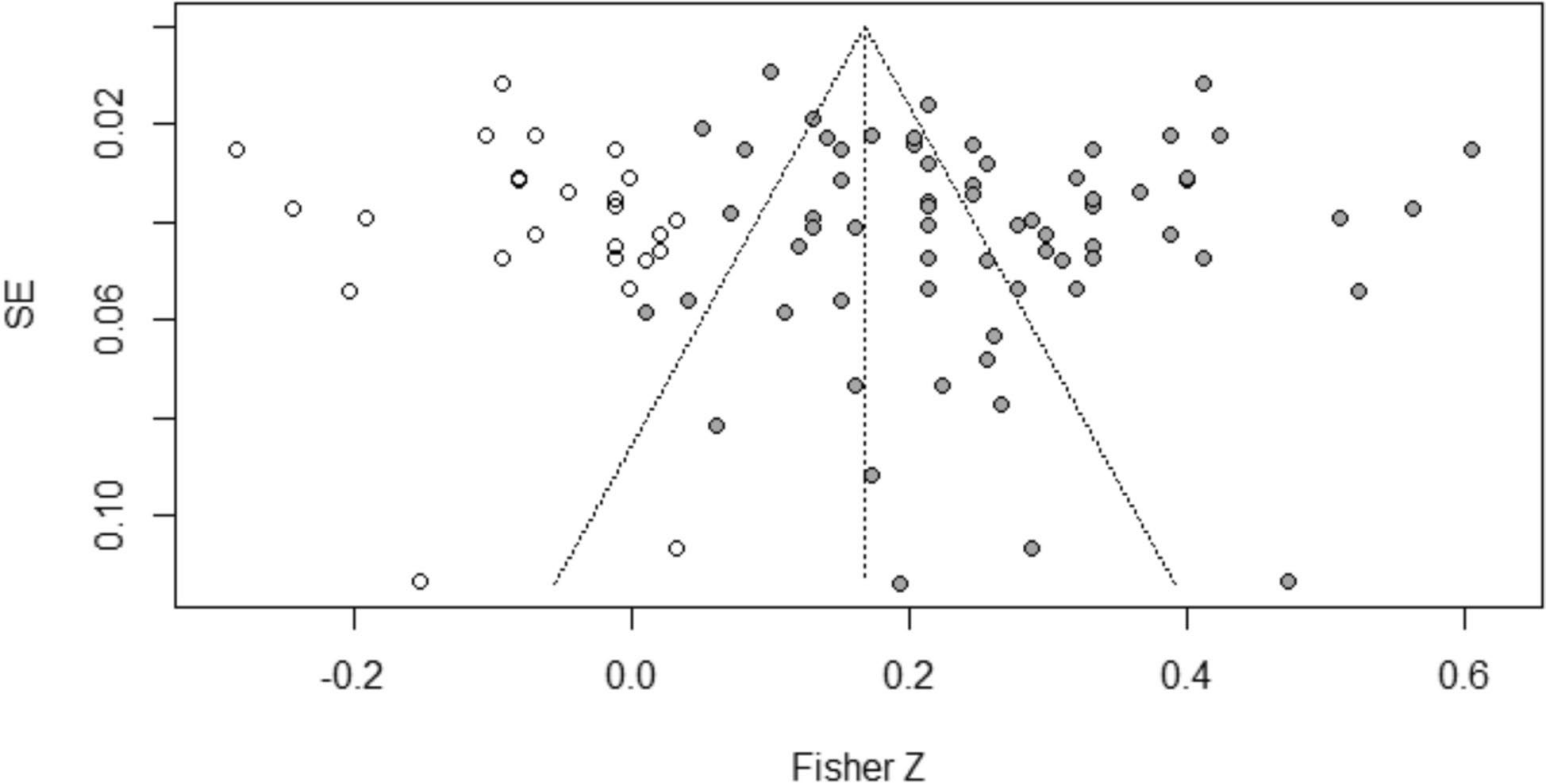
Trim-and-Fill adjusted funnel plot for the meta-analysis of parent–child conflict and childhood depression. Solid circles represent observed effect sizes; open circles represent studies imputed by the Trim-and-Fill procedure to adjust for potential publication bias

**Fig. 5:**
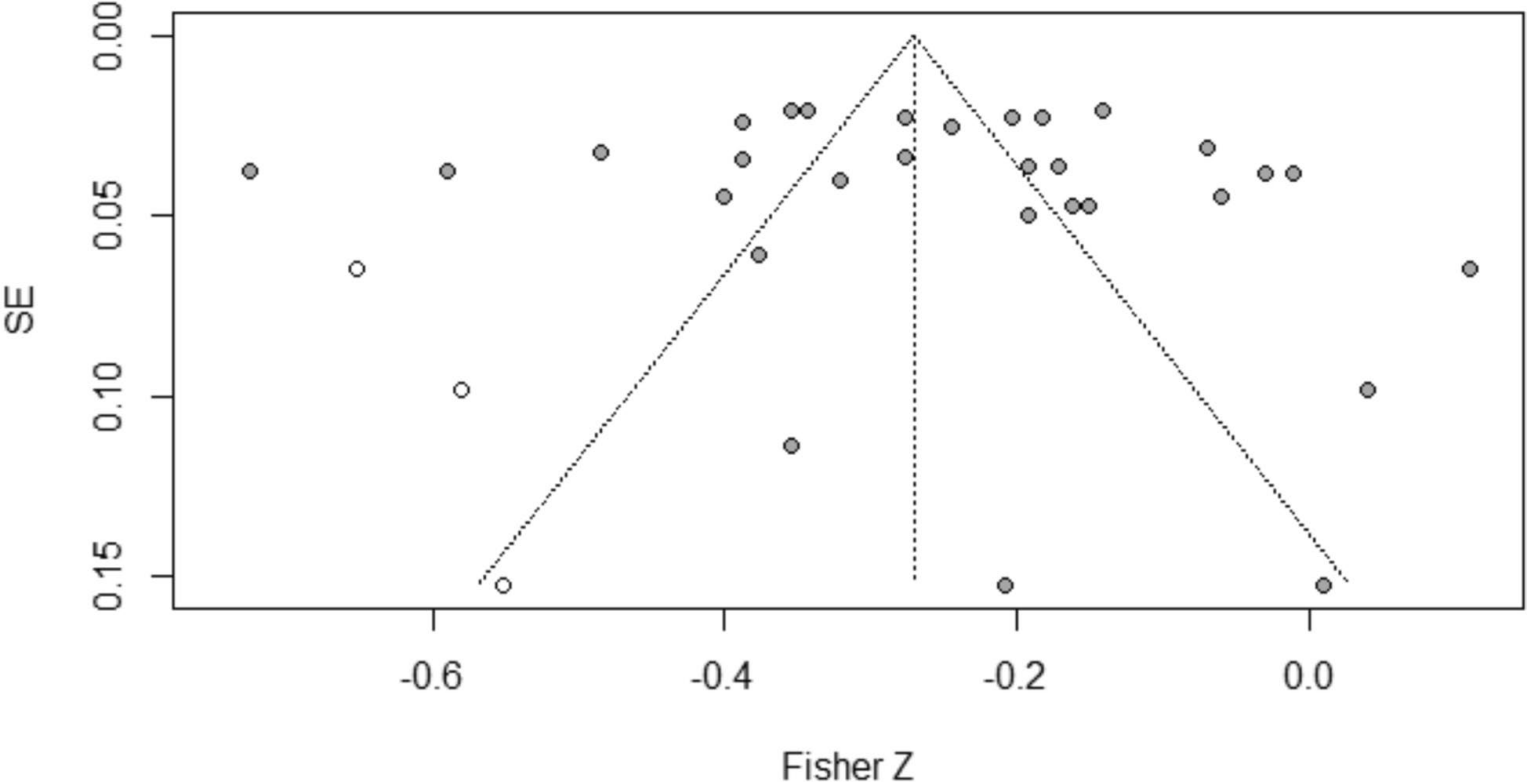
Trim-and-Fill adjusted funnel plot for the meta-analysis of parent–child closeness and childhood depression. Solid circles represent observed effect sizes; open circles represent studies imputed by the Trim-and-Fill procedure to adjust for potential publication bias

For parent–child closeness and childhood depression, Egger’s test was also non-significant (intercept = 1.38, *p* = 0.53). However, the Trim-and-Fill analysis revealed a more complex pattern, identifying 25 potentially missing studies predominantly with smaller or negative effect sizes. The adjusted effect size (*r* = 0.167, 95% CI = 0.13–0.20) represented a 33.6% reduction from the original estimate (*r* = 0.25) but remained statistically significant (*p* < 0.001). This substantial adjustment suggests that the original effect size for parent–child closeness may have been overestimated due to publication bias. Figures 4 and 5 present the Trim-and-Fill adjusted funnel plots, with observed studies (solid circles) and imputed studies (open circles).

To further assess the robustness of findings, sensitivity analyses using the leave-one-out method were conducted. When any individual study was excluded, effect sizes remained stable and significant: *r* ranged from − 0.25 to − 0.27 for parent–child conflict and depression, and from 0.23 to 0.25 for parent–child closeness and depression. These results indicate that no single study disproportionately influenced the overall findings.

Collectively, these analyses suggest that the association between parent–child conflict and childhood depression is robust to publication bias concerns. For parent–child closeness, while the core association remains significant after adjustment, the magnitude of the effect should be interpreted more cautiously given the substantial number of studies imputed by the Trim-and-Fill procedure. This differential pattern across the two dimensions of parent–child relationships highlights the importance of comprehensive bias assessment in meta-analytic research.

## Discussion

Parent–child relationships, as a central issue in child psychological development, have garnered widespread attention. Researchers have accumulated substantial evidence concerning their association with child mental health, among which the predictive effect of parent–child relationships on depression is particularly prominent. However, due to factors such as variations in measurement instruments, heterogeneity in cultural backgrounds, and the age range of samples, existing studies show discrepancies in the strength and direction of this relationship. This study integrates 63 empirical studies worldwide (totaling 97 effect sizes) through meta-analysis, representing the first systematic integration of research on parent–child relationships and child depression.

### Parent–child relationship and child depression

This study found that parent–child relationship quality exerts a moderate-strength predictive effect on children’s depression risk (*r* = 0.25 for conflict; *r* = − 0.24 for closeness). This conclusion aligns with numerous prior studies. The results support the core hypothesis of attachment theory: secure attachment promotes the development of children’s emotion regulation capabilities by providing stable emotional support and a foundation of trust, while insecure attachment may lead to emotional suppression or over-reactivity, increasing vulnerability to depression [14]. Family systems theory further elucidates that when parent–child interactions are chronically characterized by high conflict or low closeness, the rupture of family emotional bonds weakens children’s psychological resilience, facilitating the internalization of negative emotions [35, 64]. Furthermore, the stress-buffering model indicates that positive parent–child relationships can mitigate the detrimental impact of external stressful events on children’s mental health by providing emotional support and social resources [28], whereas conflictual interactions amplify the negative effects of stressors [32]. This may occur because high-quality parent–child relationships provide adolescents with emotional support and psychological security [65], thereby effectively reducing their depressive symptoms. According to interpersonal stress theory, strained parent–child relationships interfere with adolescents’ social behavioral functioning and emotion regulation abilities, increasing their susceptibility to depression [26]. Conversely, positive parent–child relationships help adolescents develop positive cognitive patterns and emotion regulation strategies, reducing their sensitivity to negative life events [45]. Therefore, this study found that higher parent–child relationship quality significantly reduces adolescents’ depression risk, further validating core tenets of existing theories and revealing that focusing on the quality of parent–child relationships is crucial for children’s mental health development.

### Moderator analysis

This study is the first to examine the moderating variables that influence the relationship between parenting and depression, and provides preliminary insights into the reasons for the inconsistent results of established studies on the relationship. The results indicated that the relationship was influenced by moderating factors such as children’s age, cultural background, and study design.

The findings indicate that child age significantly moderates the relationship between parent–child closeness and child depression, but not the relationship between parent–child conflict and child depression. Specifically, the protective effect of parent–child intimacy on child depression was characterized by dynamic changes [28, 45]. In early childhood, parent–child closeness buffers the effects of stressful events on depression by providing emotional support and secure attachment [23, 66], whereas the protective effects of parent–child closeness may work through different mechanisms as individuals’ need for autonomy increases with age, especially after entering adolescence. For example, Yan et al. [45] found that a slower decline in father-daughter closeness in mid-childhood significantly alleviated depressive symptoms in girls. This is consistent with family systems theory, which suggests that chronic conflict disrupts core attachment mechanisms regardless of age. This supports family systems theory, which posits that chronic conflict undermines attachment mechanisms irrespective of age [67]. Attachment theory further explains that conflict fosters anxious or avoidant attachment patterns by disrupting a child’s secure base, impairing emotional regulation [68, 69]. Once these patterns become internalized, they persist, influencing interpersonal interactions and emotional responses over time [70, 71]. Cross-sectional and longitudinal studies have also confirmed the ongoing association between parent–child conflict and depression from childhood through adolescence [32, 35].

The present study found that cultural context significantly moderates the relationship between parent–child conflict and depression, with collectivism exerting a greater influence than individualism. Despite changes in traditional Chinese family dynamics, parents maintain high emotional involvement, meaning conflicts may be seen as threats to hierarchy, resulting in greater psychological stress [72, 73]. Conversely, in individualistic cultures like the United States, parent–child conflict can be viewed as a sign of independence, potentially buffered by external social support. Furthermore, the emphasis on harmony in Chinese families can lead children to internalize shame from conflict, raising their risk of depression [74, 75]. Additionally, the moderating effect of sample country on the relationship between parent–child closeness and child depression was significant, with the strongest effects in the Chinese sample [48]. In this context, child closeness serves not only as emotional support but also reinforces identity and belonging, effectively mitigating depressive symptoms [57, 76]. In contrast, the Western perspective on closeness often emphasizes emotional autonomy, which may weaken its protective effects as adolescents pursue independence [77]. Notably, father involvement patterns in Chinese families also play a critical role in reducing girls’ depression risk through established emotional connections. In addition, Chinese families’ emphasis on the value of “harmony” may make it easier for children to internalize the shame associated with conflict, increasing the risk of depression [46].

The present study found significant moderating effects of study design in the association between parent–child conflict and child depression, with cross-sectional studies having significantly larger effect sizes than longitudinal studies, suggesting that the immediate effects of parent–child conflict on child depression may diminish over time. Specifically, cross-sectional studies are more likely to capture the immediate effects of parent–child conflict on children’s mood, as they often synchronize the measurement of conflict events with depressive symptoms through self-assessment instruments [48], whereas dynamic mitigation of conflict or adaptive processes in longitudinal designs may attenuate their effects [45]. Notably, the moderating effect of study design on parent–child closeness and depression did not reach a significant level, which may stem from the fact that parent–child closeness, as a stable and protective factor over time, is less affected by the way the data are collected, and both the static assessment of relationship quality in cross-sectional studies and the dynamic trajectory of closeness in longitudinal studies consistently show a sustained buffering effect on children’s depression.

This study found that publication status significantly moderated the relationship between parent–child closeness and depression. Specifically, compared to unpublished studies, published studies demonstrated a stronger negative predictive effect of parent–child closeness on depression. This finding may reflect potential publication bias in the academic publishing process, where studies with statistical significance or larger effect sizes are more likely to be accepted by journals. Furthermore, published studies typically employ more rigorous methodological designs, leading to a systematic bias in their estimation of the true effect. However, publication status failed to significantly moderate the relationship between parent–child conflict and depression. This might be because the association between parent–child conflict, as a risk factor, and depression may be subject to greater influence from contextual variables, resulting in a more dispersed distribution of effect sizes in unpublished studies.

This study found that both child gender and parental gender did not significantly moderate the association between parent–child relationships and depressive symptoms in children. This indicates that the relationship between the two has a universal transgender characteristic. The family system theory emphasizes the interdependence among family members [64], indicating that the influence of parents on their children is usually exerted through the overall functioning of the family system rather than through independent pathways.

For example, parent–child conflicts or intimate relationships may be seen by children as common features of the family system. Especially in collectivist cultures, the complementarity of parental roles [73, 78] may weaken the unique influence of parents through the mediating effect of family harmony or cohesion. With the transformation of contemporary family structures, fathers’ participation in parenting activities has significantly increased, leading to a weakening trend in the traditional complementarity of parental roles [72, 78].This phenomenon may further strengthen the integrity of the family system, making individual differences in parental behavior more easily absorbed by common family functions. Although individual studies have identified parental specific effects [28, 45, 79], these differences may be masked by cross-cultural heterogeneity or measurement limitations in meta-analyses.

This study also examined the potential moderating role of depression measurement instruments in the association between parent–child relationships and child depression. The results revealed that the instrument used to measure child depression failed to demonstrate any significant moderating effect on the influence of either parent–child closeness or parent–child conflict on child depression. This indicates that the impact of parent–child closeness and conflict on child depression exhibits stability across different measurement tools. This stability highlights the broad impact of parent–child relationships on child depression, signifying that the influence of parent–child relationships on child depression demonstrates consistency regardless of individual or contextual differences.

### Limitations and future directions

This study systematically combed similar studies on the relationship between parent–child relationship and child depression through meta-analysis, and analyzed the strength of the association between the two from the two dimensions of parent–child relationship, and found that parent–child conflict was significantly and positively associated with child depression, while parent–child intimacy was significantly and negatively associated with depression. This study is the first to quantitatively verify the influence of parent–child intimacy and conflict on children’s depression, providing cross-cultural empirical support for attachment theory and family systems theory in developmental psychology, filling the gap of inconsistency between the degree of correlation and the regulatory mechanism in previous studies, and providing a scientific intervention path to reduce the risk of children’s depression through enhancing parent–child intimacy and mitigating parent–child conflict in the practice of family education.

Limitations of this study remain: First, residual heterogeneity may exist due to diverse measurement tools, sample characteristics, and methodological designs across included studies. This complicates the interpretation of effect size consistency. Future research should develop cross-culturally harmonized core indicators of parent–child relationships using mixed-methods designs. This approach can help control measurement bias and enhance ecological validity. Second, despite using funnel plots, Egger’s test, and the Trim-and-Fill method to address heterogeneity, other unconsidered moderating variables may still exist. Future studies should explore additional moderating variables such as socioeconomic status, family structure, parenting styles, and adolescent individual differences (e.g., temperament, gender). Investigating these factors could provide a more nuanced understanding of how parent–child relationships impact adolescent depression. Third, the inclusion of only Chinese and U.S. data in the moderated analyses, along with insufficient sample sizes from other regions, may limit the cross-cultural generalizability of the findings. Most studies were conducted in Western and East Asian cultures. Future research should include more diverse cultural contexts, particularly from low-income countries and non-Western societies. This can improve the theoretical framework of family socialization in cross-cultural contexts and help identify culturally specific protective and risk factors. Finally, while this study differentiated between father-child and mother–child relationships, the moderating effect of parental roles was not significant. This may be due to most studies not independently reporting the effect of parental roles on parent–child relationship quality. Future research should refine the parental role variable and expand samples to include special family structures (e.g., single father families). This can help reveal the differential moderating pathways of parental gender and parenting division of labor.

## Conclusions

This study systematically integrated empirical research on parent–child relationships and child depression through meta-analytic techniques, revealing the intrinsic mechanisms and boundary conditions of their associations. Main effects tests indicated that parent–child conflict was significantly positively associated with child depression, while parent–child closeness showed a significant negative association. Moderating effect analyses found that child age, cultural background, research design, and publication status played a moderating role in the parent–child and depression relationships. Specifically, cultural background and study design had significant moderating effects on the association between parent–child conflict and depression, reflecting the potential influence of differences in conflict expression and temporal dynamics in cross-cultural contexts on the results; whereas in the association between parent–child closeness and depression, children’s age, cultural background, and publication status of the literature showed a moderating effect, reflecting the characteristics of the children’s developmental stage and the differentiation of cultural values on the emotional support Needs. Notably, child gender, parental role, and the measurement instrument did not show significant moderating effects, suggesting cross-gender generalizability and measurement robustness of the mechanisms of parent–child relationship effects on child depression.

## Data Availability

The datasets used or analyzed during the current study are available from the corresponding author upon reasonable request.
